# Tomato *Mi*-gene Resistance-Breaking Populations of *Meloidogyne* Show Variable Reproduction on Susceptible and Resistant Crop Cultivars

**DOI:** 10.2478/jofnem-2023-0043

**Published:** 2023-10-16

**Authors:** A. T. Ploeg, C. S. Stoddard, T. A. Turini, J. J. Nunez, E. M. Miyao, S. A. Subbotin

**Affiliations:** Department of Nematology, University of California Riverside, 3401 Watkins Drive, Riverside, CA 92521; UCCE Merced & Madera Counties, 2145 Wardrobe Ave, Merced, CA 9534; UCCE Fresno County, 550 E. Shaw Avenue, Suite 210-B, Fresno, CA 93710; UCCE Kern County (retired), 1031 South Mount Vernon Avenue Bakersfield, CA 93307; UCCE Yolo, Solano & Sacramento Counties (retired), 70 Cottonwood St Woodland, CA 95695; CDFA, 3294 Meadowview Road, Sacramento, CA 95832, USA

**Keywords:** cotton, green bean, *Meloidogyne incognita*, *Meloidogyne javanica*, pepper, resistance breaking, root-knot nematodes, *Solanum lycopersicum*, sweetpotato, tomato

## Abstract

Sixteen *Meloidogyne* isolates from tomato fields in California grown with resistant cultivars were multiplied on resistant tomato in a greenhouse. Of these resistance-breaking isolates, one was identified as *M. javanica*, and all others as *M. incognita.* The reproduction of the *M. javanica* isolate and four *M. incognita* isolates on six resistant tomato cultivars and on susceptible and resistant cultivars of pepper, sweetpotato, green bean, cotton, and cowpea was evaluated and compared to an avirulent *M. incognita* population in greenhouse pot trials. On resistant tomato cultivars, there were minor but significant differences between the resistance-breaking *Meloidogyne* isolates and between the different tomato cultivars. Of the other resistant crop cultivars, pepper was resistant to all isolates and green bean to all *M. incognita* isolates, while cotton and cowpea allowed reproduction of one of the resistance-breaking *M. incognita* isolates. The resistant sweetpotato cv. Bonita behaved like resistant tomato, allowing reproduction of all five resistance-breaking isolates but not of the avirulent *M. incognita*. Our results showed that variability exists among resistance-breaking *Meloidogyne* isolates, and that isolates overcoming resistance in tomato may also be virulent on resistant sweetpotato.

The USA is the largest producer of processing tomatoes (*Solanum lycopersicum*), accounting for 34% of worldwide production. Approximately 95% of the US acreage is in California (Anonymous, 2019), representing a value of over $1 billion. Over the last five years, California acreage has remained relatively constant at 230,000 acres, with an average yield of 45–50 tons/acre (USDA-NASS 2021 report). Root-knot nematodes (RKN: *Meloidogyne* spp.), particularly the Southern root-knot nematode (*M. incognita*) and the Javanese root-knot nematode (*M. javanica*), are the most important nematodes limiting yields of tomato worldwide and in California (Roberts, 1992; Williamson, 1998; Kiewnick et al., 2009; Wesemael et al., 2011). Annual crop losses in processing tomato production in California have been estimated between 10–20% (Koenning et al., 1999). To mitigate crop losses by nematodes, two strategies are primarily used by California growers: soil fumigants such as metam-sodium, metam-potassium, and 1,3-dichloropropene, which in 2021 accounted for 19% of the total pesticide active ingredient used in this crop (Anonymous, 2021a, 2021b), and growing nematode-resistant cultivars. The latter strategy has been successfully employed for many years, and almost all current cultivars are resistant to three main RKN species: *M. incognita*, *M. javanica*, and *M. arenaria* (Roberts, 1992; Williamson, 1998). The resistance in all available commercial cultivars is based on the presence of the single dominant *Mi*-1 gene, which was introgressed into a commercial cultivar using embryo rescue from the wild tomato relative *Solanum peruvianum* (Gilbert and McGuire, 1956; Kaloshian et al., 1996; Williamson, 1998). Although the *Mi*-1 gene has been used for over 70 years, it remains a very effective nematode management strategy (Castagnone-Sereno et al., 2007). However, the occurrence of *Meloidogyne* populations that can reproduce on resistant cultivars has been reported from tomato-growing areas worldwide, and the frequency of resistance breaking appears to be increasing (Sikora et al., 1973; Netscher, 1977; Berthou et al., 1989; Prot, 1984; Eddaoudi et al., 1997; Ornat et al., 2001; Djian-Caporalino et al., 2011). In the USA, the occurrence of resistance-breaking populations of *M. incognita* was initially reported from California (Kaloshian et al., 1996) and recently also from Georgia (Hajihassani et al., 2022). While the appearance of resistance-breaking populations has been linked to repeated exposure to resistant tomatoes (Netscher, 1977; Viglierchio, 1978; Castagnone-Sereno et al., 1993; Noling, 2000; Meher et al., 2009), such populations have also been isolated in fields with no history of resistant tomato crops (Riggs and Winstead, 1959; Kaloshian et al., 1996; Eddaoudi et al., 1997; Ornat et al., 2001; Tzortzakakis et al., 2005; Hajihassani et al., 2022). Hajihassani et al., (2022) reported differences in the degree of virulence between resistance-breaking populations on the same resistant tomato cultivar, but others (Castagnone-Sereno et al., 1994) reported no differences between different resistance-breaking populations. Hajihassani et al. (2022) found no differences between three resistant tomato cultivars when exposed to the same resistance-breaking population, but Jacquet et al. (2005) did report a significant cultivar effect in trials where eight different *Mi*-gene-carrying tomato hybrids were exposed to virulent *M. incognita* populations.

To slow down the selection of virulent populations, Castagnone-Sereno et al. (2007) suggested alternating resistant with susceptible tomato varieties as a practical management strategy. Djian-Caporalino et al. (2011) reported that an *M. incognita* isolate virulent on resistant tomato failed to reproduce on resistant pepper and, conversely, that an *M. incognita* isolate virulent on resistant pepper did not reproduce on resistant tomato. They concluded that virulence is highly specific to the resistance for which selection occurred, and that *Meloidogyne* isolates that are virulent on one resistant crop are definitely not virulent on another resistant crop. Thus, they suggested that rotating crops with different resistance genes could be a useful tactic to avoid the rapid selection and build-up of virulent RKN isolates (Djian-Caporalino et al., 2011).

The goals of this study were to determine the variability between RKN populations isolated from resistant tomato fields in California, to determine the variability between different *Mi*-gene processing tomato cultivars when exposed to such virulent RKN populations, and to determine if such populations are indeed avirulent on other RKN-resistant crop cultivars. For the latter, nematode reproduction and symptom development on *M. incognita*-susceptible and -resistant cultivars of pepper, sweetpotato, green bean, cowpea, and cotton were assessed in greenhouse pot trials.

## Materials and Methods

*Origin, isolation, and propagation of Meloidogyne populations:* Over the course of two growing seasons, roots of plants from 16 processing tomato fields in California with *Mi*-gene resistant cultivars exhibiting stunted growth were dug up. Their roots were examined for root galling symptoms, and they were sent to the Department of Nematology at UC Riverside when symptomatic (Table 1). The roots were then washed free of soil, cut into approximately 1-cm-long pieces, and placed in a misting chamber (Niblack and Hussey, 1985) for 5 days for nematode extraction. The suspensions were then examined at ×40 magnification using a dissecting microscope to check for the presence of *Meloidogyne* second-stage juveniles (J2). If they were present, these suspensions were added to a 6-week-old tomato cv. Celebrity plant with the *Mi*-gene (Bayer/Seminis, St. Louis, MO) grown in steam-sterilized sand (93% sand, 4% silt, 3% clay, pH 7.1) in a 3.8-liter pot in a greenhouse. Plants were grown under natural light at soil temperatures between 21–26 °C, watered daily through an automated drip system, and fertilized with 10g of a slow-release fertilizer (Osmocote 17-6-10, Scotts, Marysville, OH) one week after transplanting. Eight weeks later, the roots of these plants were washed and examined, and if galling was present, a single egg mass was removed from the roots under a dissecting microscope at ×10 magnification and added to a 4-week-old cv. Celebrity tomato seedling growing in a 55-ml plastic cone (Stuewe and Sons, Corvallis, OR) with the same steam-sterilized sand in a greenhouse. Plants were carefully removed from the cone four weeks later and transferred to a 3.8-liter pot. They were grown for another 6 weeks, and then nematodes were extracted from the roots in a misting chamber as described above. Nematodes from these roots were used as inoculum for further multiplication on tomato cv. Celebrity plants, and roots from these cv. Celebrity plants were used as a source of inoculum for experiments and for nematode identification.

**Table 1.** Origin of 16 field-collected Mi-gene resistant processing tomato root samples used for nematode isolation.

**Nematode population number**	**Origin County**	**Field-grown tomato cultivar**
1	Yolo	Heinz5508
2	San Joaquin	SUN6366
3	Yolo	Heinz5508
4	Kern	CXD187
5	Yolo	Heinz5508
6	Kern	Heinz5208
7	Kern	Heinz5208
8	Fresno	UG19406
9	Fresno	SUN6366
10	Fresno	SUN6366
11	Yolo	Heinz5508
12	Yolo	Heinz5508
13	Solano	Heinz5508
14	Colusa	N6416
15	Sutter	H1310
16	Colusa	HM3888

*Nematode identification:* RKN females were excised from galled tomato cv. Celebrity roots, and perineal patterns from five females per root system were cut for morphological identification (Riggs, 1990). Patterns were examined under ×500 magnification using a Leitz DMR compound light microscope and based on the patterns, nematodes were identified to species level (Eisenback, 1985). In addition to morphological identification, five populations that were used for further experiments were also identified through PCR. Of those, four that were identified as *M. incognita* based on perineal patterns were used to amplify and sequence the *nad5* mtDNA gene fragment. Three μl of extracted DNA were transferred to a 0.2-ml Eppendorf tube containing: 10 μl DreamTaq Green PCR Master Mix (2×) (Thermo Fisher Scientific, Waltham, MA), 10 μl water and 0.15 μl of each primer (1.0 μg/μl). Forward NAD5F2 (5′-TAT TTT TTG TTT GAG ATA TAT TAG-3′) and reverse NAD5R1 (5′-CGT GAA TCT TGA TTT TCC ATT TTT-3′) primers as described by Janssen et al. (2016) were used in PCR. The PCR amplification profile consisted of 4 min at 94°C, followed by 40 cycles of 1 min at 94°C, 1 min at 45°C, and 1 min 30 sec at 72°C, with a final extension at 72°C for 10 min. Two μl of the PCR product were run on a 1% TAE-buffered agarose gel (100 V, 40 min). PCR products were purified using QIAquick PCR Purification Kit (Qiagen, Hilden, Germany) and sequenced directly. Sequencing was performed by Genewiz (CA, USA). New sequences were compared with reference sequences using Blastn. Sequence was submitted in the Genbank under accession number OM418740. To confirm the identity of one population that was identified based on perineal patterns as *M. javanica*, J2 were used in a species-specific assay for this species, as described by Dong et al. (2001), using primers Fjav 5′-CCT TAA TGT CAA CAC TAG AGC C-3′ and Rjav 5′-GGC CTT AAC CGA CAA TTA GA-3′.

*Nematode multiplication and effects on susceptible and resistant plant varieties Tomato:* Five nematode populations that originated from single-egg-mass cultures were randomly selected for further experiments. In a greenhouse pot trial, the multiplication and symptom expression of these populations on a range of *Mi*-gene-carrying resistant processing tomato cultivars was determined. Tomato cultivars used in the trial were DRI319, H8504, H5608, HM3887, and N6366. These tomato cultivars were selected based on recommendations by the California Tomato Research Institute (CTRI) as processing tomato cultivars currently grown in California (Anonymous, 2021). Seed of these cultivars was also kindly provided by CTRI. As controls, the resistant *Mi*-gene-carrying fresh-market cultivar Celebrity (Bayer/Seminis, St. Louis, MO) and the susceptible cultivar Daniela (Osborne Quality Seeds, Mt. Vernon, WA) were included. Also, a “standard” avirulent *M. incognita* race 3 population (*Mi*-3) originally isolated from cotton in the San Joaquin Valley, California that had been maintained on susceptible tomato in our greenhouse for several years was included as an internal control. For the trial, tomatoes were seeded in seedling trays with potting mix (Sunshine Mix 5, Sungro, Vancouver, BC, Canada) and placed in a greenhouse. Three weeks after emergence, plants were carefully removed from the seedling trays and transplanted into 1-liter white plastic cups filled with steam-sterilized sand. Ten days after transplanting, each plant was inoculated with 1,000 RKN J2 in 9 ml water by adding 3 ml each of the suspension to three 3-cm-deep holes around the base of each plant. For inoculation, pots receiving the same RKN population were placed on a bench and separated from the other pots. This was repeated for each RKN population.

The trial was set up according to a completely randomized design with five replicates (a total of 6 RKN populations × 7 tomato cultivars × 5 replicates = 210 pots). The pots were placed on a greenhouse bench with a 40-cm spacing between the pots. Plants were watered with a slow water drip to avoid the splashing associated with automated drip systems, and fertilized with 17–6-10 controlled release fertilizer (Scotts-Sierra Horticultural Products Co, Marysville, OH). Six weeks after inoculation, the plants were removed from the pots, and the roots were washed free of soil, weighed, and rated for severity of root galling (0–10 scale: 0 = no galling, 10 = 100% of root system galled). Roots were then placed in a mist chamber for 7 days to extract RKN J2. The suspensions coming off the roots were collected, and the number of J2 from each root system was counted under a dissecting microscope at ×40 magnification. Nematode reproduction is expressed as the reproduction factor RF (Pf/Pi = number of J2 per root system at harvest/number of J2 inoculated). The complete experiment was repeated once (trial 1 and 2).

Differences between the populations, tomato cultivars, and their interactive effects were analyzed statistically using R software (R Core Team, 2022). The repeated trials were considered random effects. Prior to analysis, J2 counts were transformed by x^1^ = log10 (x + 1) before analysis. Where treatment effects on J2 infestation levels were significant, they were separated with Fisher's protected Least Significant Difference test at the 95% confidence level. Differences in galling levels were analyzed using the non-parametric Kruskal-Wallis test, also at the 95% confidence level. In the experiments with tomato, the *Mi*-3 population was not included in the statistical analysis, as it was included only as an internal standard to confirm the resistant nature of the different tomato cultivars, and we were primarily interested in differences among the resistance-breaking nematode populations.

In a series of subsequent experiments, the same nematode populations were used as inoculum on susceptible and resistant crop cultivars. The experiments were done as with tomato, with two replicated trials. Statistical analysis was as with the tomato trial. In each of the experiments, the *Mi*-gene-carrying resistant tomato cv. Celebrity was included as an internal control to confirm the virulence of the populations on tomato, but not included in the statistical analysis.

*Pepper:* The *M. incognita*-susceptible pepper (*Capsicum annuum*) cv. Baron (Seminis, Oxnard, CA) and the *M. incognita*-resistant cv. Carolina Wonder (Reimer Seeds, Saint Leonard, MD) were used. Experiments were as with tomato, but time between seeding and transplanting was 4 weeks.

*Sweet potato and green bean:* Sweetpotato (*Ipomea batatas*) stem cuttings of *M. incognita-*susceptible cv. O’Henry and *M. incognita*-resistant cv. Bonita were kindly donated by the California Sweetpotato Council. The cuttings were planted in vermiculite and placed in a misting chamber in a greenhouse. Plants were carefully removed from the vermiculite 10 days later, and cuttings exhibiting root growth were planted in 1-liter plastic cups with steam-sterilized sand, as with the tomato experiments. One week later, these plants were inoculated with the nematode populations. At harvest, some plants had developed small storage roots, but these were not included for J2 extraction. In the same experiment, the *M. incognita*-susceptible green bean (*Phaseolus vulgaris*) cv. BlueLake (Harris Seeds, Rochester, NY) and the *M. incognita*-resistant cv. Nemasnap (USDA-GRIN) were seeded directly into 1-liter plastic cups with steam-sterilized sand. Bean plants were inoculated with the nematode populations 10 days after emergence. All plants in the trial were inoculated simultaneously.

*Cotton and cowpea: M. incognita*-susceptible cotton (*Gossypium hirsutum*) cv. SJ2 (originally obtained from California Planting Cotton Seed Distributers and propagated in our greenhouse), the *M. incognita*-resistant cotton cv. DP 1474NR B2XF (seed kindly provided by Dr. Wheeler, Texas A&M), the *M. incognita*-susceptible cowpea (*Vigna unguiculata*) cv. CB46 Null-1 and the *M. incognita*-resistant cv. CB46 (seed kindly provided by Dr. Huynh, Dept. Nematology, UC Riverside) were seeded directly into 1-liter plastic cups with steam-sterilized sand. All plants were inoculated with the nematode populations simultaneously (cowpea: 14 days after emergence, cotton: 21 days after emergence).

## Results

*Nematode identification:* Perineal patterns of females from all nematode populations were consistent with those described for *M. incognita*, except for population 4, which had perineal patterns with typical double lateral lines and was identified as *M. javanica*. PCR-based identification on the five populations used for further experiments (including population 4 identified as *M. javanica*) confirmed the results from the morphological identification.

*Nematode multiplication and ef fects on susceptible and resistant plant varieties Tomato:* Statistical analysis showed that both “tomato cultivar” and “RKN population” had a significant effect (*P ≤ 0.01*) on nematode reproduction and root-galling. The interaction between these two factors, however, was not significant (*P > 0.05*). The avirulent *Mi*-3 population, included as a control, behaved as expected, with a high reproduction on the susceptible cv. Daniela and very few nematodes recovered from the resistant tomato cultivars. There were significant differences among the five resistance-breaking populations, with populations 3, 10, and 11 reproducing at higher levels than populations 4 and 12 (Table 2). There were also significant differences in the susceptibility of the different tomato cultivars towards the resistance-breaking populations.

**Table 2.** Average (n=10) reproduction factor (RF) and galling index (scale 0 – 10 = 0 = no galling and 10 = 100% of root system galled) on seven different tomato cultivars inoculated with five resistance-breaking root-knot nematode (*Meloidogyne*) populations (1,000 second-stage juveniles/pot) and an avirulent *M. incognita* race 3 population in a greenhouse pot trial.

	**Tomato cultivar**								

	**Daniela (S^a^)**	**Celebrity (R^a^)**	**H5608 (R^a^)**	**DRI319 (R^a^)**	**N6366 (R^a^)**	**HM3887 (R^a^)**	**H8504 (R^a^)**		
Reproduction factor (RF)									
Nematode population^b^									
*Mi*-3 control	8.5	0.1	0.5	0.2	0.1	0.2	0.2		
Resistance-breaking							average		
3 (*Mi*)	4.1	3.7	11.8	12.9	4.9	5.6	2.9	6.6	A^d^
4 (*Mj*)	3.1	1.6	9.6	7.9	2.0	2.4	1.0	3.9	B
10 (*Mi*)	4.4	6.8	5.1	10.7	8.7	3.1	4.2	6.1	A
11 (*Mi*)	6.6	4.7	17.0	11.1	4.3	6.1	4.1	7.7	A
12 (*Mi*)	2.5	3.5	4.1	3.1	4.2	4.7	3.8	3.7	B
average	4.1 b^c^	4.0 b	9.5 a	9.1 a	4.8 ab	4.4 b	3.2 b		
Galling index									
Nematode population^b^									
*Mi*-3 control	6.2	0.7	0.7	0.3	0.5	0.4	0.4		
Resistance-breaking							average		
3 (*Mi*)	6.4	5.5	7.0	6.8	6.8	6.6	5.6	6.4	A^d^
4 (*Mj*)	4.0	5.0	6.0	6.2	6.3	6.2	4.9	5.5	BC
10 (*Mi*)	5.5	6.2	7.1	6.6	6.5	6.4	6.0	6.3	AB
11 (*Mi*)	6.1	4.9	6.4	6.9	6.5	7.4	6.4	6.4	A
12 (*Mi*)	4.3	3.5	4.8	5.0	4.3	5.4	4.3	4.5	C
average	5.3 ab^c^	5.0 b	6.3 a	6.3 a	6.1 ab	6.4 a	5.4 ab		

a (S): root-knot nematode susceptible cultivar, (R): root-knot nematode resistant cultivar.

b The control *Mi*-3 population is shown for comparison and was not included in the statistical analysis. (*Mi*) indicates a population identified as *M. incognita*, (*Mj*) indicates a population identified as *M. javanica*.

c Different lowercase letters indicate significant differences between between different tomato varieties at the 95% confidence level. Reproduction Factor: Fisher's protected LSD-test. Statistical analysis performed on log(*x*+1)-transformed data, non-transformed data shown. Galling index: Kruskal-Wallis test.

d Different uppercase letters indicate significant differences between the different resistance-breaking *Meloidogyne* populations at the 95% confidence level.

Although all cultivars allowed a strong multiplication (RF > 3) of the resistance-breaking populations, cultivars H5608 and DRI319 were even more susceptible than the susceptible Daniela and the *Mi-*gene-carrying resistant cultivars Celebrity, HM3887, and HH8504 (Table 2). The severity of root-galling on tomato largely reflected the nematode multiplication data. The avirulent *Mi*-3 control population caused severe galling on the susceptible cv. Daniela and very minor galling on the resistant tomato cultivars. Populations 3, 10, and 11 also caused the most severe galling, while galling resulting from populations 4 and 12 was less dramatic. All cultivars exhibited moderately high galling after inoculation with the resistance-breaking populations, although minor but significant differences existed. Galling on the *Mi-*gene-carrying resistant cv. Celebrity was less than on the *Mi-*gene-carrying resistant cultivars H5608, DRI319, and HM3887 (Table 2). Fresh root weights of the tomatoes were not significantly different between the different tomato cultivars (*P > 0.05*; data not shown).

*Pepper:* In the trials with peppers, the interactive effect of “pepper cultivar” × “RKN population” on nematode reproduction and root galling was significant (*P ≤ 0.01*). Differences between the nematode populations within each pepper cultivar are thus shown. The tomato cv. Celebrity, included as a control, confirmed the avirulent nature of the *Mi*-3 population and the virulent nature of the five resistance-breaking populations on tomato. There was a significant difference among the RKN populations (*P ≤ 0.01*) in nematode reproduction on pepper cv. Baron (Table 3). This cultivar was highly susceptible to all populations, except to population 4, which failed to reproduce or cause any galling on this cultivar. The *M. incognita*-resistant pepper cv. Carolina Wonder was highly resistant to all populations, and differences among the populations were not significant (*P > 0.05*). Only very minimal galling was observed on the roots of cv. Carolina Wonder plants, although population 12 caused slightly more galling on this cultivar than most other populations (Table 3).

**Table 3.** Average (n=10) reproduction factor (RF) and root galling index (scale 0 – 10 = 0 = no galling and 10 = 100% of root system galled) on two different pepper cultivars inoculated with six root-knot nematode (*Meloidogyne*) populations (1,000 second-stage juveniles/pot) in a greenhouse pot trial.

	**Plant cultivar**		

	**Tomato Celebrity^a^ (R^b^)**	**Pepper Baron (S^b^)**	**Pepper Carolina Wonder (R^b^)**
Reproduction factor (RF)			
Nematode population^c^			
*Mi*-3 control	0.3	17.5 a^d^	0.0 a^d^
Resistance-breaking			
3 (*Mi*)	22.2	21.7 a	0.0 a
4 (*Mj*)	27.6	0.0 b	0.1 a
10 (*Mi*)	25.5	15.9 a	0.0 a
11 (*Mi*)	35.8	17.6 a	0.0 a
12 (*Mi*)	29.1	27.0 a	0.0 a
Galling index			
Nematode population^c^			
*Mi*-3 control	0.3	3.5 cd^e^	0.0 b^e^
Resistance-breaking			
3 (*Mi*)	7.3	5.2 ab	0.0 b
4 (*Mj*)	5.8	0.0 e	0.1 ab
10 (*Mi*)	6.5	4.5 bc	0.0 b
11 (*Mi*)	6.7	3.1 d	0.0 b
12 (*Mi*)	7.0	6.0 a	0.3 a

a Tomato cv. Celebrity was included as an internal control and not included in the statistical analysis.

b (S): root-knot nematode susceptible cultivar, (R): root-knot nematode resistant cultivar.

c (*Mi*) indicates a population identified as *M. incognita*, (*Mj*) indicates a population identified as *M. javanica*.

d Different letters within a column indicate significant differences in reproduction factor between RKN populations at the 95% confidence level, Fisher's protected LSD-test. Statistical analysis performed on log(*x*+1)-transformed data, non-transformed data shown.

e Different letters within a column indicate significant differences in galling index between RKN populations at the 95% confidence level, Kruskal-Wallis test.

*Sweetpotato and green bean:* In the trials with sweetpotato and green bean, results from the internal standard – the resistant tomato cv. Celebrity – were as before, showing strong resistance against the *Mi*-3 population, while being highly susceptible to the five virulent (resistance-breaking) populations. The interactive effect of crop cultivar × RKN population on nematode reproduction and root-galling was highly significant (*P ≤ 0.01*). The *M. incognita-*susceptible sweetpotato cv. O’Henry and the *M. incognita*-susceptible bean cv. BlueLake were highly susceptible to all RKN populations, and galling on the roots of these plants at harvest was severe, regardless of the RKN population that was used as inoculum (Table 4).

**Table 4.** Average (n=10) reproduction factor (RF) and root galling index (scale 0 – 10 = 0 = no galling and 10 = 100% of root system galled) on two sweetpotato and two green bean cultivars inoculated with six root-knot nematode (*Meloidogyne*) populations (1,000 second-stage juveniles/pot) in a greenhouse pot trial.

	**Crop cultivar**				

	**Tomato Celebrity^a^ (R^2^)**	**Sweetpotato O’Henry (S^2^)**	**Sweetpotato Bonita (R^2^)**	**Green bean BlueLake (S^2^)**	**Green bean Nemasnap (R^2^)**
Reproduction factor (RF)					
Nematode population^c^					
*Mi*-3 control	0.4	30.4 a^d^	0.7 b^d^	42.9 a^d^	0.1 c^d^
Resistance-breaking					
3 (*Mi*)	55.2	52.1 a	5.2 a	47.1 a	0.1 c
4 (*Mj*)	35.7	49.6 a	4.1 a	52.5 a	6.6 a
10 (*Mi*)	62.3	28.4 a	2.8 a	45.8 a	0.1 c
11 (*Mi*)	61.4	54.4 a	3.2 a	37.2 a	0.3 bc
12 (*Mi*)	48.9	47.3 a	3.7 a	39.9 a	0.4 b
Galling index					
Nematode population^c^					
*Mi*-3 control	0.2	5.1 a^e^	0.6 a^e^	6.2 a^e^	0.3 b^e^
Resistance-breaking					
3 (*Mi*)	6.0	6.1 a	1.1 a	5.7 a	0.0 b
4 (*Mj*)	5.3	6.2 a	0.9 a	6.0 a	4.8 a
10 (*Mi*)	5.8	5.0 a	1.2 a	5.9 a	0.5 b
11 (*Mi*)	6.5	5.1 a	1.2 a	5.3 a	0.2 b
12 (*Mi*)	5.9	5.6 a	1.5 a	5.6 a	0.2 b

a Tomato cv. Celebrity was included as an internal control and not included in the statistical analysis.

b (S): root-knot nematode susceptible cultivar, (R): root-knot nematode resistant cultivar.

c (*Mi*) indicates a population identified as *M. incognita*, (*Mj*) indicates a population identified as *M. javanica*.

d Different letters within a column indicate significant differences in reproduction factor between RKN populations at the 95% confidence level, Fisher's protected LSD-test. Statistical analysis performed on log(*x*+1)-transformed data, non-transformed data shown.

e Different letters within a column indicate significant differences in galling index between RKN populations at the 95% confidence level, Kruskal-Wallis test.

Significant differences among the RKN populations existed both in the *M. incognita-*resistant sweetpotato cv. Bonita and the green bean cv. NemaSnap. Sweetpotato cv. Bonita was resistant to the *Mi*-3 control population (RF = 0.7), but susceptible (RF = 2.8–5.2) to the five populations known to be able to break resistance in tomato. This difference between *Mi*-3 and the five resistance-breaking populations was not reflected in the severity of root-galling on sweetpotato cv. Bonita, as this was minor with all RKN populations. The green bean cv. NemaSnap was highly susceptible to population 4, but highly resistant to the other five populations. This corresponded with the severity of root-galling on cv. NemaSnap after inoculation with the different nematode populations (Table 4).

*Cotton and cowpea:* Finally, in the trial with cotton and cowpea, results on the internal control tomato cv. Celebrity were again as expected, and the interactive effect of crop cultivar × RKN population on nematode reproduction and root-galling was again highly significant (*P ≤ 0.01*). The *M. incognita*-susceptible cotton cv. SJ2 was an excellent host for *Mi-*3 and population 12, a maintenance host for population 4, and highly resistant to populations 3, 10, and 11. Susceptible cowpea cv. CB46 Null-1 was an excellent host for all six RKN populations, although reproduction of population 3 (RF = 16.4) was significantly lower compared to the other five populations. Galling on the susceptible cotton cultivar corresponded with results regarding nematode reproduction, and galling on the susceptible cowpea cultivar was high and did not differ among the RKN populations. Resistant cotton cv. DP 1474NR B2XF prevented a nematode increase (RF < 1) of all populations except population 12, which increased twofold. Galling on the roots of the resistant cotton cultivar was very minor with all RKN populations. Two RKN populations, 4 and 12, were able to increase more than 10-fold on *M. incognita-*resistant cowpea cv. CB46, whereas this cultivar was resistant to the other four populations (RF < 1). Root-galling on cowpea cv. CB46 reflected these results with regards to nematode reproduction (Table 5).

**Table 5.** Average (n=10) reproduction factor (RF) and root galling index (scale 0 – 10 = 0 = no galling and 10 = 100% of root system galled) on two cotton and two cowpea cultivars inoculated with six root-knot nematode (*Meloidogyne*) populations (1,000 second-stage juveniles/pot) in a greenhouse pot trial.

	**Crop ultivar**				

	**Tomato Celebrity^a^ (R^b^)**	**Cotton SJ2 (S^b^)**	**Cotton DP 1474NR B2XF (R^b^)**	**Cowpea CB46 Null-1 (S^b^)**	**Cowpea CB46 (R^b^)**
Reproduction factor (RF)					
Nematode population^c^					
*Mi*-3 control	0.5	14.5 a^d^	0.1 bc^d^	205.0 a^d^	0.1 b^d^
Resistance-breaking					
3 (*Mi*)	111.4	0.1 c	0.0 d	16.2 b	0.9 b
4 (*Mj*)	88.8	1.4 b	0.6 ab	91.1 a	10.4 a
10 (*Mi*)	84.2	0.0 c	0.3 bc	76.9 a	0.1 b
11 (*Mi*)	128.9	0.1 c	0.1 cd	143.7 a	0.2 b
12 (*Mi*)	85.2	19.7 a	2.0 a	93.8 a	11.4 a
Galling index					
Nematode population^c^					
*Mi*-3 control	0.4	3.0 a^e^	0.5 ab^e^	6.8 a^e^	0.1 b^e^
Resistance-breaking					
3 (*Mi*)	7.9	0.1 c	0.0 b	6.0 a	0.9 b
4 (*Mj*)	7.8	1.6 b	0.6 ab	6.1 a	2.5 a
10 (*Mi*)	6.9	0.3 c	0.4 ab	6.7 a	0.4 b
11 (*Mi*)	7.4	0.2 c	0.1 ab	7.2 a	0.6 b
12 (*Mi*)	8.0	2.6 ab	0.9 a	5.6 a	2.9 a

a Tomato cv. Celebrity was included as an internal control and not included in the statistical analysis.

b (S): root-knot nematode susceptible cultivar, (R): root-knot nematode resistant cultivar.

c (*Mi*) indicates a population identified as *M. incognita*, (*Mj*) indicates a population identified as *M. javanica*.

d Different letters within a column indicate significant differences in reproduction factor between RKN populations at the 95% confidence level, Fisher's protected LSD-test. Statistical analysis performed on log(*x*+1)-transformed data, non-transformed data shown.

e Different letters within a column indicate significant differences in galling index between RKN populations at the 95% confidence level, Kruskal-Wallis test.

## Discussion

Fifteen of the 16 populations isolated from fields planted with *Mi*-gene-carrying resistant processing tomato in California were identified as *M. incognita,* and one was identified as *M. javanica.* It is unknown if *M. incognita* is more common in areas where processing tomato is grown, or if resistance-breaking occurs more easily in *M. incognita*. Recent data on the occurrence of RKN in California are not available, but in surveys by Siddiqi et al. (1973), 19 detections of *M. incognita* and 14 of *M. javanica* were reported associated with tomato, which suggests *M. incognita* is only slightly more common than *M. javanica* on California tomato. Resistance-breaking by *M. javanica* was reported in California by Williamson (1998), but that population was obtained by repeated greenhouse culturing of an originally avirulent population on tomato with *Mi*-mediated resistance. In the only other report on resistance-breaking RKN populations from the USA outside of California, which is from Georgia, all populations were identified as *M. incognita* (Hajihassani et al., 2022). Thus, this is the first report of a field-isolated population of *M. javanica* on *Mi*-gene-carrying resistant tomato in the USA. Resistance-breaking field populations of *M. javanica* have been reported in several other countries, however (Tzortzakakis and Gowen, 1996; Eddaoudi et al., 1997).

Although all five resistance-breaking populations reproduced well on the tomato cultivars evaluated in this study, there were differences. Populations 4 and 12 had significantly lower RF values than the other three. Differences in reproduction among four resistance-breaking *M. incognita* populations on *Mi*-gene-carrying resistant tomato were also reported by Hajihassani et al. (2022). They speculated that this might be due to genetic diversity among the different nematode populations tested, and this may also explain our results. In addition to differences between the RKN populations, there were also differences among the *Mi*-gene-carrying resistant tomato cultivars, with two cultivars, H5608 and DRI319, being particularly susceptible. It can be assumed that the resistance of all cultivars used in this study is based on the same single dominant *Mi*-gene (Williamson, 1998).

Jacquet et al. (2005), evaluating a range of tomato *Mi*-gene-carrying cultivars, obtained similar cultivar effects, and concluded that differences in the genetic background of these cultivars influenced the level of resistance towards resistance-breaking RKN populations. Previous research (Castagnone-Sereno et al., 2007) showed that resistance-breaking in tomato by *M. incognita* is associated with a loss in reproductive fitness, and although we only included one avirulent *M. incognita* population in our study, our results are similar, as the RF of the avirulent population on the susceptible tomato cv. Daniela, was higher than of the four resistance-breaking populations.

The susceptible pepper cv. Baron was an excellent host for all *M. incognita* populations, but not for the *M. javanica* population. Pepper is generally considered a non-host for *M. javanica*, and is in fact used as a differential host to distinguish between *M. incognita* and *M. javanica* (Eisenback et al., 1981). The *N*-gene-mediated resistance in pepper cv. Carolina Wonder was highly effective in preventing nematode reproduction in all RKN populations. Thus, the populations breaking resistance in tomato were not able to overcome *N*-gene-mediated resistance in pepper. This is similar to results from Djian-Caporalino et al. (2011), who also reported that RKN resistance in pepper was maintained when challenged with populations virulent on *Mi*-gene-mediated resistant tomato.

Sweetpotato cv. O’Henry was equally and highly susceptible to all RKN populations. On the RKN-resistant sweetpotato cv. Bonita there was a clear and significant separation in nematode reproduction between the avirulent *Mi*-3 control population and the five resistance-breaking populations. Whereas the RF of the former population was 0.7, i.e., a decrease in nematode levels, the five resistance-breaking populations all multiplied more than twofold (RF = 2.8–5.2) on sweetpotato cv. Bonita. Thus, sweetpotato cv. Bonita can be considered resistant to the avirulent *Mi*-3 population, but susceptible to the five resistance-breaking populations. Galling on cv. Bonita roots was minor, however, and did not differ among the RKN populations.

In previous field studies on sweetpotato by Roberts and Scheuerman (1984) and by us (Ploeg, unpublished) a large RKN population increase in soil was observed even after one cropping cycle with a RKN-resistant cultivar. However, Roberts and Scheuerman (1984) reported that nematode-resistant sweetpotato cultivars did not develop typical RKN symptoms on the storage roots (bumpiness and cracking), and we found very large differences between the number of RKN eggs extracted from field-grown sweetpotato storage roots between susceptible cv. O’Henry (117 eggs/g storage root) and those found in resistant cv. Bonita (0.1 eggs/g storage root) (Ploeg, unpublished). Thus, it appears that RKN resistance in sweetpotato is primarily related to preventing nematode reproduction and symptom development on the storage roots (tubers), rather than nematode reproduction in the true feeder roots. The genetic basis for RKN resistance in cv. Bonita is unknown, but La Bonte et al. (2011) reported cv. Bonita to be highly resistant to *Mi*-3 in greenhouse studies.

Green bean cv. Blue Lake was an excellent host for all RKN populations, while the resistant cv. NemaSnap was highly resistant to all *M. incognita* populations, yet susceptible to *M. javanica* (population 4). Resistance of cv. NemaSnap to *M. incognita* was reported previously (Wyatt et al., 1983; Mullin et al., 1991), but information on *M. javanica* is limited to cv. NemaSnap being moderately resistant to a mixed population of *M. javanica* and *M. incognita*.

The host status of the susceptible cotton cv. SJ2 differed depending on the RKN population. It was a host for *Mi*-3, *M. incognita* population 12 and *M. javanica* (population 4). *Meloidogyne incognita* populations can be categorized as belonging to four distinct races, with populations able to reproduce on cotton belonging to race 3 or 4 (Barker et al., 1985). The race 3 designation of the control population corresponds with this result. Population 12 should be designated as a race 3 or race 4 population.

We did not include tobacco in our test, a differential host for *M. incognita* race 3 versus race 4. Resistant cotton cv. DP 1474NR B2XF prevented a nematode increase in all populations except of *M. incognita* population 12, which on average doubled on this cultivar.

The susceptible cowpea cv. CB46 Null-1 was an excellent host for all RKN populations. The resistant cowpea cv. CB46 was a good host for *M. javanica* (population 4), and for *M. incognita* population 12. The resistance in cowpea cv. CB46 is based on the *Rk* gene, and although resistance to *M. incognita* is generally strong, variability in the effectiveness of its resistance towards different *M. incognita* populations has been documented (Huynh et al., 2016). Furthermore, *Rk*-gene-mediated resistance in cowpea is generally less effective against avirulent populations of *M. javanica* than of *M. incognita* (Huynh et al., 2016).

Overall, it can be concluded that RKN populations able to break *Mi*-gene resistance in tomato are common throughout tomato-growing areas in California. We observed minor variability in the reproductive potential among these populations on resistant tomato cultivars, as well as some variability in resistance towards them among different *Mi*-gene-carrying tomato cultivars. The statement by Djian-Caporalino et al. (2011) that “RKN isolates virulent on one resistant crop are definitely not virulent on a different crop” may not be always true, as in our study only those populations virulent on *Mi*-gene-carrying resistant tomato were able to reproduce on the resistant sweetpotato cv. Bonita. This clear separation between the avirulent *Mi*-3 population and the resistance-breaking populations was not observed on the other crops tested.

This study also demonstrates the variability in virulence among the resistance-breaking populations when inoculated onto other resistant crop cultivars, as significant differences in reproduction were observed among the RKN populations on resistant bean, cotton and cowpea. The pepper cv. Carolina Wonder was the only crop cultivar that was highly resistant to all the RKN populations used in this study, and this crop may have potential for use in rotation with tomato to reduce levels of resistance-breaking RKN populations. This study shows that relying solely on one strategy, such as the use of host plant resistance to manage RKN, may lose its effectiveness in the long run. It also stresses the importance of alternating or integrating different management strategies, such as chemical, biological, or cultural control, in order to maintain the required reductions in population levels over time.
